# GDM Women’s Pre-Pregnancy Overweight/Obesity and Gestational Weight Gain on Offspring Overweight Status

**DOI:** 10.1371/journal.pone.0129536

**Published:** 2015-06-22

**Authors:** Junhong Leng, Weiqin Li, Shuang Zhang, Huikun Liu, Leishen Wang, Gongshu Liu, Nan Li, Leanne M. Redman, Andrea A. Baccarelli, Lifang Hou, Gang Hu

**Affiliations:** 1 Project Office, Tianjin Women’s and Children’s Health Center, Tianjin, China; 2 Chronic Disease Epidemiology Laboratory, Pennington Biomedical Research Center, Baton Rouge, Louisiana, United States of America; 3 Department of Epidemiology, Harvard School of Public Health, Boston, Massachusetts, United States of America; 4 Department of Environmental Health, Harvard School of Public Health, Boston, Massachusetts, United States of America; 5 Department of Preventive Medicine, Feinberg School of Medicine, Northwestern University, Chicago, Illinois, United States of America; University of São Paulo, BRAZIL

## Abstract

**Objectives:**

To examine the association of maternal pre-pregnancy body mass index (BMI) and gestational weight gain (GWG) with anthropometry in the offspring of mothers with gestational diabetes mellitus (GDM).

**Methods:**

We performed a retrospective cohort study in 1263 GDM mother-child pairs. General linear models and Logistic regression models were used to assess the single and joint associations of maternal pre-pregnancy BMI (normal weight, overweight, and obesity) and GWG (inadequate, adequate and excessive GWG) with anthropometry and overweight status in the offspring from birth to 1-5 years old.

**Results:**

Maternal pre-pregnancy BMI and GWG were positively associated with birth weight for gestational age Z score and birth weight for length for gestational age Z score at birth, and weight for age Z score, length/height for age Z score, and weight for length/height Z score at of 1-5 years old offspring. Maternal pre-pregnancy overweight, obesity, and excessive GWG were associated with increased risks of large for gestational age [ORs 95% CIs = 1.87 (1.37-2.55), 2.98 (1.89-4.69), and 2.93 (2.07-4.13), respectively] and macrosomia [ORs 95% CIs = 2.06 (1.50-2.84), 2.89 (1.78-4.70), and 2.84 (1.98-4.06), respectively] at birth and childhood overweight at 1-5 years old [ORs 95% CIs = 1.26 (0.92-1.73), 1.96 (1.24-3.09), and 1.59 (1.15-2.21), respectively].

**Conclusions:**

Offspring born to GDM mothers with pre-pregnancy overweight/obesity or excessive GWG were associated with increased risks of large for gestational age and macrosomia at birth, and childhood overweight at 1-5 years old, compared with those born to GDM mothers with pre-pregnancy normal weight and adequate GWG.

## Introduction

Obesity has progressed in a few decades from a public health footnote in developed countries to a top-priority international issue, affecting all socioeconomic classes and age groups including children younger than 5 years old [1, 2]. The prevalence of overweight or obesity has increased substantially in children and adolescents: 23.8% (22.9–24.7) of boys and 22.6% (21.7–23.6) of girls in the developed countries, and 12.9% (12.3–13.5) of boys and 13.4% (13.0–13.9) of girls in the developing countries in 2013 [3]. Recent studies have shown that excessive weight gain and/or overweight in the first several years of life are associated with increased risks of subsequent obesity and unfavorable cardiometabolic outcomes in childhood, adolescence, and adulthood [4–6]. Identifying risk factors in early prenatal and postnatal life related to later obesity may lead to developing early intervention strategies for primordial obesity prevention [4].

Maternal pre-pregnancy overweight and obesity have also increased nowadays [7, 8]. Moreover, approximately half of women in 2009 gained more weight than recommended by the Institute of Medicine (IOM) guidelines worldwide [8]. Both maternal pre-pregnancy overweight/obesity and excessive gestational weight gain (GWG) would increase the risk of poor maternal and fetal outcomes, including macrosomia and large for gestational age of neonates [9–14]. A US longitudinal cohort study of children 2–12 years of age found that maternal pre-pregnancy body mass index (BMI) was associated with both children early onset and late onset overweight, while excessive maternal GWG was associated with children early onset overweight [15]. However, most of the studies focusing on the association of maternal pre-pregnancy BMI and GWG with childhood overweight status were conducted among general women, and there is no direct evidence on the association of maternal pre-pregnancy BMI and GWG of GDM mothers on their offspring overweight status at 1–5 years old. Studies have found that offspring of mothers exposed to GDM were at increased risk of neonatal adiposity and childhood obesity [16–18]. GDM is one of the most common complications in pregnancy, affecting 2–10% of the pregnancies in the US [19], and Asian women compared with other racial/ethnic groups in the US have a higher risk for GDM [20–23]. The aim of the present study was to examine the single and joint associations of maternal pre-pregnancy BMI and GWG according to the IOM guidelines with the offspring aged 1–5 years of GDM mothers.

## Materials and Methods

### Tianjin GDM screening project

Tianjin is the fourth largest city in Northern China. All pregnant women who live in six urban districts have participated in the universal screening for GDM since 1999 [24] by using the World Health Organization (WHO)’s GDM criteria [25]. Following the WHO’s GDM screening criteria, all pregnant women at 26–30 gestational weeks participate in a 1-hour oral glucose tolerance test (OGTT) with 50-g glucose load. Women who had a glucose reading ≥7.8 mmol/l were invited to undergo a 2-hour OGTT with a 75-g glucose load at the Tianjin Women’s and Children’s Health Center. All women confirming either diabetes (plasma fasting glucose ≥7 mmol/l or 2-hour glucose ≥11.1 mmol/l) or impaired glucose tolerance (IGT) (2-hour glucose ≥7.8 and <11.1 mmol/l) were regarded as having GDM [24]. From December 1998 to December 2009, a total of 128,125 pregnant women took part in the GDM screening program and 6,247 were diagnosed with GDM [26]. The average proportion of screened pregnancies was over 91% during 1999–2009 [24].

### Study samples

The sampling methods have been described previously in detail [26]. Since we had set up a good health care registration system for GDM mothers’ health and contact information, all pregnant women in six urban districts diagnosed with GDM between 2005 and 2009 (N = 4,644) were invited to participate in a postpartum baseline survey for the *Tianjin Gestational Diabetes Mellitus Prevention Program* from August 2009 to July 2011. Finally, 1263 GDM women and their children had completed the baseline survey for the TGDMPP from August 2009 to July 2011 (participation rate 27%) [26–29]. Between the returned and unreturned GDM women, there were no differences at 26–30 gestational weeks OGTT test by age (28.9 vs. 28.7 years), 2-hour glucose (9.23 vs. 9.16 mmol/l), fasting glucose (5.34 vs. 5.34 mmol/l), and prevalence of IGT (90.7% vs. 91.8%) and diabetes (9.3% vs. 8.2%). The study was approved by the Human Subjects Committee of the Tianjin Women’s and Children’s Health Center, informed consent was obtained for each participant.

### Examinations

At baseline survey, all GDM mothers and their children completed a self-administered questionnaire and underwent a physical examination that included anthropometric and blood pressure measurements, a 2-hour glucose 75g OGTT (mothers only), and a fasting blood draw at the Tianjin Women’s and Children’s Health Center. Health workers from the Tianjin Women’s and Children’s Health Center collected and checked the completed questionnaire and also finished measurements. All health workers were intensively trained in meetings and in practical sessions. The questionnaire included questions on the mother’s socio-demographics (age, marital status, education, income, and occupation); history of GDM (values of fasting and 2-hour glucose in the OGTT at 26–30 gestational weeks from the Tianjin Women’s and Children’s Health Center GDM diagnosis and treatment register system); family history of chronic diseases; medical history (hypertension, diabetes, and hypercholesterolemia); pregnancy outcomes (pre-pregnancy weight, weight gain during pregnancy, and number of children); dietary habits (a self-administered food frequency questionnaire (FFQ) to measure the frequency and quantity of intake of 33 major food groups and beverages during the past year) [30]; alcohol intake; smoking habits; and physical activity [31].

We also asked the GDM children’s parents in advance to bring the child’s birth certificate and filled in a self-administered questionnaire about the child’s birth date, sex, gestational weeks of birth, birth weight, birth recumbent length, and Apgar score (above questions related to birth were copied from birth certificate); as well as the mode and duration of infant feeding (exclusive breast feeding, mixed breast and formula feeding, weaned from breast feeding, and exclusive formula feeding), health characteristics (history of illness status and current health status), dietary habits (usual habits of eating breakfast, lunch, and dinner, usual frequency of intake of vegetables, fruits, sugar-sweetened beverages, and fast food), and other lifestyle habits (duration of usual sleep, and television or computer viewing). This questionnaire has been used in a longitudinal study in the same area of Tianjin [32, 33].

For GDM mothers, body weight and height were measured using the standardized protocol according to the WHO MONICA project [34]. Children’s body weight was measured with a beam balance scale with participants wearing light indoor clothing without shoes, and height was measured by a stadiometer. Weight was measured to the nearest 0.1 kg and height to the nearest 0.1 cm. BMI was calculated by dividing weight in kilograms by the square of height in meters. Maternal pre-pregnancy BMI was categorized as normal weight (BMI < 24 kg/m^2^), overweight (BMI 24–27.9 kg/m^2^) and obesity (BMI ≥28 kg/m^2^) using the standard of Working Group on Obesity in China [35]. Adequacy of GWG was classified according to the Chinese maternal pre-pregnancy BMI classification standard and the 2009 IOM GWG recommendations: 12.5–18 kg (BMI <18.5 kg/m^2^), 11.5–16 kg (BMI 18.5–23.9 kg/m^2^), 7–11.5 kg (BMI 24.0–27.9 kg/m^2^), and 5–9 kg (BMI ≥28 kg/m^2^) (S1 Table) [36]. Inadequacy of GWG was defined as below adequacy of GWG and excessive of GWG was defined as above the adequacy of GWG. The 2009 IOM GWG recommendation has been widely used in Chinese pregnant women [37].

Z scores for birth weight for gestational age and birth weight for length for gestational age were calculated using our own study population means and standard deviations (n = 57,454) in 2009–2011 [38]. A large-for-gestational-age infant was defined as an infant having a standardized birth weight >90^th^ percentile. Macrosomia was defined as birth weight ≥4000 grams. At baseline (1–5 years of offspring age), z scores for weight for age, height for age, weight for length and BMI for age, and childhood overweight were calculated based on the standards for the WHO growth reference [39]. Children’s overweight was defined as a BMI more than or equal to the 85^th^ percentiles for age and gender using the WHO BMI growth reference (≥1.035 Z score) [39].

### Statistical analyses

The general characteristics of both GDM mothers and offspring according to different categories of maternal pre-pregnancy BMI and GWG were compared using one-way ANOVA and chi-square test. General Linear Models were used to compare the differences in Z scores for birth weight for gestational age and birth weight for length for gestational age, Z scores for weight for age, height for age, weight for height and BMI for age at baseline survey, and changes in Z scores for weight for age and weight for height from birth to baseline (1–5 years of offspring age) according to different categories of maternal pre-pregnancy BMI and GWG. Logistic regression was used to estimate the odd ratios (ORs) and 95% confidence intervals (95% CIs) of large for gestational age and macrosomia at birth, and childhood overweight at baseline survey with maternal pre-pregnancy BMI and GWG categories. We set up two models: Model 1, adjusted for variables with significant differences according to maternal pre-pregnancy BMI and gestational weight gain categories, including maternal age, family history of diabetes, education, income, gestational diabetes treatment during pregnancy, gestational weeks of birth, and infant feeding; Model 2, adjusted for variables in Model 1 and also for variables at birth, including birth weight for gestational age Z-score or birth weight for length for gestational age Z score. All statistical analyses were performed with SAS for Windows, version 9.3 (SAS Institute, Cary, NC).

## Results

Maternal and offspring’s general characteristics were presented in Table 1. Of the 1263 GDM mothers, the prevalence of pre-pregnancy normal weight, overweight and obesity were 65.2%, 26.5% and 8.3%, and the prevalence of inadequate, adequate and excessive GWG were 12.4%, 31.2% and 56.5%, respectively.

**Table 1:** Characteristics of 1263 gestational diabetes mellitus mother-child pairs in Tianjin Gestational Diabetes Mellitus Prevention Program.

	Pre-pregnancy BMI (kg/m^2^)			P	IOM categories ^a^			P
	<24	24–27.9	≥28		Inadequate	Adequate	Excessive	
No. of subjects	823	335	105		156	394	713	
**Maternal characteristics**								
Age (years)	32.3 (3.5)	32.4 (3.7)	32.5 (3.6)	0.83	32.9 (3.5)	32.7 (3.6)	32.1 (3.5)	0.002
Pre-pregnancy BMI	21.2 (1.8)	25.6 (1.1)	30.3 (2.0)	<0.001	21.7 (2.7)	22.0 (2.9)	24.0 (3.4)	<0.001
Fasting glucose at 26–30 gestational weeks (mmol/l)	5.25 (0.8)	5.49 (0.9)	5.99 (1.7)	<0.001	5.55 (1.3)	5.31 (0.9)	5.38 (0.9)	0.032
2-hour glucose at 26–30 gestational weeks (mmol/l)	6.69 (2.2)	7.48 (2.5)	8.80 (3.4)	<0.001	7.36 (3.1)	7.03 (2.5)	7.04 (2.4)	0.32
Gestational diabetes treatment during pregnancy (%)				<0.001				0.099
No	14.8	14.6	9.5		14.1	10.7	16.4	
Lifestyle intervention	82.4	79.4	78.1		80.1	84.5	79.7	
Insulin	2.8	6.0	12.4		5.8	4.8	3.9	
Gestational weight gain								
Means (kg)	16.8 (5.8)	17.4 (6.1)	14.6 (6.5)	<0.001	8.82 (2.3)	13.5 (2.5)	20.4 (5.1)	<0.001
Categories (%)				<0.001				
Inadequate	23.0	18.8	9.5		-	-	-	
Adequate	40.2	30.7	28.6		-	-	-	
Excessive	36.8	50.4	61.9		-	-	-	
Current smoking (%)	1.7	1.8	4.8	0.10	1.9	2.5	4.5	0.12
Education (%)				<0.001				<0.001
<13 years	19.5	25.4	37.1		22.4	16.5	25.8	
13–16 years	72.1	67.8	61.0		69.2	72.5	68.9	
≥16 years	8.4	6.9	1.9		8.3	10.9	5.3	
Family income (yuan/month)				<0.001				0.20
<5000	23.5	34.6	36.2		29.5	23.9	29.1	
5000–8000	37.7	34.6	38.1		34.6	36.5	37.6	
≥8000	38.8	30.7	25.7		35.9	39.6	33.3	
Family history of diabetes (%)	34.3	39.0	38.1	0.27	36.4	32.7	37.5	0.29
**Offspring characteristics**								
*Newborn*								
Gestational weeks of birth (wks)	39.1 (1.4)	38.9 (1.6)	38.6 (1.7)	0.005	38.9 (1.5)	39.1 (1.5)	39.0 (1.5)	0.20
Sex (men %)	53.5	51.6	54.3	0.82	57.7	48.5	54.6	0.70
Birth weight (g)	3501 (493)	3608 (569)	3695 (574)	<0.001	3273 (499)	3445 (463)	3662 (529)	<0.001
Birth recumbent length (cm)	50.7 (2.0)	51.0 (2.3)	51.0 (1.8)	0.022	50.2 (2.1)	50.5 (1.9)	51.1 (2.1)	<0.001
*At baseline survey of 1–5 years old*								
Age (years)	2.28 (0.9)	2.30 (0.9)	2.24 (0.9)	0.81	2.21 (0.9)	2.24 (0.8)	2.33 (0.9)	0.14
Weight (kg)	13.3 (2.7)	13.7 (2.9)	14.1 (3.6)	0.005	13.1 (2.8)	13.1 (2.6)	13.8 (2.9)	<0.001
Height (cm)	90.4 (8.4)	90.9 (8.3)	90.8 (9.1)	0.69	89.6 (8.7)	89.8 (8.2)	91.2 (8.5)	0.014
Body mass index (kg/m^2^)	16.2 (1.4)	16.5 (1.5)	16.9 (1.8)	<0.001	16.1 (1.3)	16.1 (1.4)	16.5 (1.6)	<0.001
Breast feeding (%)				0.069				0.003
Exclusive breast feeding ≥6 months	41.3	40.1	41.0		29.5	45.7	40.9	
Exclusive breast feeding <6 months	2.1	2.1	1.0		47.4	42.1	42.0	
Mixed feeding	44.5	38.3	42.9		3.2	2.3	1.5	
Exclusive formula feeding	12.2	19.5	15.2		19.9	9.9	15.6	

Baseline characteristics represent mean (SD) or percentage.

^a^ IOM categories: Inadequate (1): <12.5 kg (pre-pregnancy BMI <18.5 kg/m^2^), <11.5 kg (BMI 18.5–23.9 kg/m^2^), <7 kg (BMI 24.0–27.9 kg/m^2^), and <5 kg (BMI >28 kg/m^2^); Adequate (1): 12.5–18 kg (BMI <18.5 kg/m^2^), 11.5–16 kg (BMI 18.5–23.9 kg/m^2^), 7–11.5 kg (BMI 24.0–27.9 kg/m^2^), and 5–9 kg (BMI >28 kg/m^2^); Excessive (1): >18 kg (BMI <18.5 kg/m^2^), >16 kg (BMI 18.5–23.9 kg/m^2^), >11.5 kg (BMI 24.0–27.9 kg/m^2^), and >9 kg (BMI >28 kg/m^2^), according to the Chinese maternal pre-pregnancy BMI classification standard and the 2009 IOM GWG recommendations.

**Fig 1:**
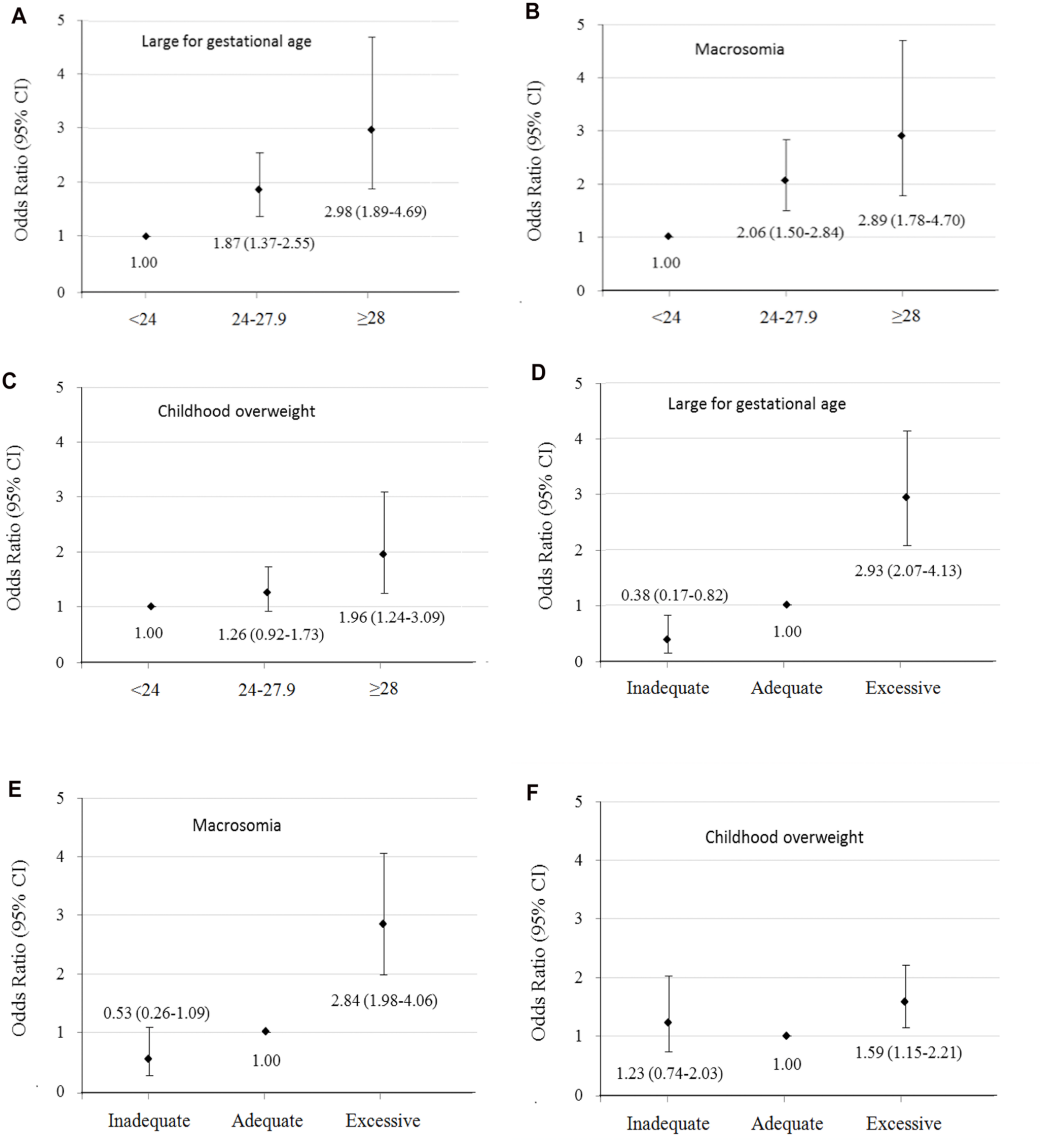
Odds ratios (95% CIs) of large for gestational age and macrosomia at birth and overweight at 1–5 years old according to maternal pre-pregnancy body mass index and gestational weight gain categories.

Maternal pre-pregnancy BMI and GWG were positively associated with birth weight for gestational age Z score (S2 Table) and birth weight for length for gestational age Z score of neonates (Table 2). Compared to offspring born to mothers with pre-pregnancy normal weight or adequate GWG, offspring born to GDM mothers with pre-pregnancy overweight/obesity or excessive GWG had larger mean values of z scores for birth weight for gestational age and birth weight for length for gestational age (Model 1). Maternal pre-pregnancy overweight, obesity, and excessive GWG were associated with increased risks of large for gestational age [ORs 95% CIs = 1.87 (1.37–2.55), 2.98 (1.89–4.69), and 2.93 (2.07–4.13), respectively] and macrosomia [ORs 95% CIs = 2.06 (1.50–2.84), 2.89 (1.78–4.70), and 2.84 (1.98–4.06), respectively] at birth, compared with maternal pre-pregnancy normal weight and adequate GWG (Fig 1).

**Table 2:** Neonatal and childhood major outcomes at birth and 1–5 years old according to maternal pre-pregnancy body mass index and gestational weight gain categories.

	Pre-pregnancy BMI (kg/m^2^)			P_overall_	IOM categories			P_overall_
	<24 (Group A)	24–27.9 (Group B)	≥28 (Group C)		Inadequate (Group D)	Adequate (Group E)	Excessive (Group F)	
No. of subjects	823	335	105		156	394	713	
**At birth** ^**e**^								
Birth weight for length for gestational age Z score	0.09 (0.09)	0.38 (0.10) *	0.69 (0.14) ^#^ ^^^	<0.001	-0.32 (0.12) ^&^	0.04 (0.10)	0.55 (0.09) ^†^ ^‡^	<0.001
**At 1–5 years old**								
Weight for length/height Z score								
Model 1 ^a^	0.38 (0.08)	0.60 (0.09) *	0.84 (0.12) ^#^ ^^^	<0.001	0.33 (0.11)	0.35 (0.09)	0.65 (0.08) ^†^ ^‡^	<0.001
Model 2 ^b^	0.43 (0.08)	0.60 (0.09) *	0.78 (0.12) ^#^	<0.001	0.45 (0.11)	0.41 (0.09)	0.61 (0.08) ^†^ ^‡^	<0.001
Body mass index for age Z score								
Model 1 ^a^	0.34 (0.08)	0.55 (0.09) *	0.78 (0.13) ^#^ ^^^	<0.001	0.30 (0.11)	0.30 (0.09)	0.59 (0.09) ^†^ ^‡^	<0.001
Model 2 ^b^	0.38 (0.08)	0.54 (0.09) *	0.73 (0.12) ^#^	<0.001	0.40 (0.11)	0.35 (0.09)	0.56 (0.08) ^†^	<0.001
Change in weight for height Z score from birth to 1–5 years old								
Model 1 ^a^	0.29 (0.11)	0.22 (0.12)	0.15 (0.17)	0.53	0.65 (0.14) ^&^	0.31 (0.12)	0.10 (0.11) ^†^ ^‡^	<0.001
Model 2 ^b^	0.08 (0.08)	0.25 (0.09) *	0.42 (0.12) ^#^	<0.001	0.09 (0.11)	0.05 (0.09)	0.26 (0.08) ^†^	<0.001

Data represent mean (SE), or percentage.

^a^, Adjusted for maternal age, family history of diabetes, education, family income, gestational diabetes treatment during pregnancy, gestational weeks of birth and infant feeding.

^b^, Adjusted for above variables and also birth weight for length for gestational age Z score.

* P <0.05 for Groups of A and B;

^#^ P<0.05 for groups of A and C;

^^^ P<0.05 for groups of B and C;

^&^ P for groups of D and E;

^†^ P <0.05 for Groups of E and F;

^‡^ P<0.05 for groups of D and F.

Maternal pre-pregnancy BMI and GWG were positively associated with weight for age Z score, length/height for age Z score (S2 Table), and weight for length/height Z score at of 1–5 years old offspring (Table 2). After further adjustment for birth weight for gestational age Z-score or birth weight for length for gestational age Z score (Model 2), offspring born to GDM mothers with pre-pregnancy overweight, obesity, and excessive GWG had increased risks of overweight at 1–5 years old [ORs 95% CIs = 1.26 (0.92–1.73), 1.96 (1.24–3.09), and 1.59 (1.15–2.21), respectively], compared with those born to GDM mothers with pre-pregnancy normal weight and adequate GWG (Fig 1).

When maternal pre-pregnancy BMI and GWG were examined as continuous variables, ORs (95% CIs) of large for gestational age, macrosomia and childhood overweight were 1.14 (1.09–1.19), 1.17 (1.12–1.22) and 1.07 (1.03–1.19) with pre-pregnancy BMI, and 1.09 (1.07–1.12), 1.09 (1.06–1.12) and 1.03 (1.01–1.06) with GWG, respectively.

The subgroup analyses of associations of large for gestational age, macrosomia and childhood overweight risks with pre-pregnancy obesity and excessive GWG were presented in Table 3. When stratified according to maternal GDM category during pregnancy, the associations with pre-pregnancy obesity and excessive GWG tended to be stronger among offspring born to mothers with pregnant Type 2 diabetes than those born to mothers with pregnant impaired glucose tolerance. When stratified into two groups according to the median of offspring age, the association of childhood overweight with maternal pre-pregnancy obesity tended to be larger among older children, while the association of childhood overweight with maternal excessive GWG tended to be smaller among older children.

**Table 3:** Odds ratios (95% CIs) of large for gestational age and macrosomia at birth and overweight at 1–5 years old stratified by maternal GDM category during pregnancy and offspring age.

Odds ratios (95% CIs)	Pre-pregnancy BMI (kg/m^2^)			P_overall_	IOM categories			P_overall_
	<24	24–27.9	≥28		Inadequate	Adequate	Excessive	
**Maternal GDM status during pregnancy**								
*Impaired glucose tolerance*	761	297	88		142	353	651	
Large for gestational age ^a^	1.00	2.01 (1.45–2.78)	2.72 (1.65–4.48)	<0.001	0.43 (0.20–0.94)	1.00	2.93 (2.03–4.23)	<0.001
Macrosomia ^a^	1.00	2.26 (1.61–3.16)	2.76 (1.63–4.70)	<0.001	0.52 (0.25–1.12)	1.00	2.78 (1.90–4.06)	<0.001
Overweight ^b^	1.00	1.23 (0.87–1.72)	2.13 (1.30–3.50)	0.011	1.36 (0.80–2.32)	1.00	1.60 (1.12–2.27)	<0.001
*Type 2 diabetes*	62	38	17		14	41	62	
Large for gestational age ^a^	1.00	0.93 (0.30–2.87)	4.16 (1.17–14.8)	0.059	—	1.00	4.67 (1.43–15.3)	0.039
Macrosomia ^a^	1.00	0.83 (0.27–2.57)	2.66 (0.73–9.76)	0.24	0.87 (0.08–10.0)	1.00	5.05 (1.51–16.9)	0.019
Overweight ^b^	1.00	1.52 (0.58–3.99)	1.85 (0.54–6.35)	0.52	0.71 (0.14–3.56)	1.00	2.25 (0.82–6.13)	0.15
**Offspring age** ^**c**^								
*1*.*0–2*.*09 years old*	415	163	52		87	208	335	
Overweight ^b^	1.00	1.34 (0.85–2.13)	1.41 (0.69–2.88)	0.37	1.26 (0.61–2.60)	1.00	2.01 (1.24–3.25)	0.015
*2*.*1–5*.*0 years old*	408	172	53		69	186	378	
Overweight ^b^	1.00	1.17 (0.75–1.82)	2.77 (1.47–5.22)	0.007	1.43 (0.70–2.93)	1.00	1.44 (0.90–2.29)	0.30

^a^, Adjusted for maternal age, family history of diabetes, education, family income, gestational diabetes treatment during pregnancy, gestational weeks of birth and infant feeding.

^b^, Adjusted for above variables and also birth weight for length for gestational age Z score.

^c^, The median of offspring age was 2.1 years old.

**Fig 2:**
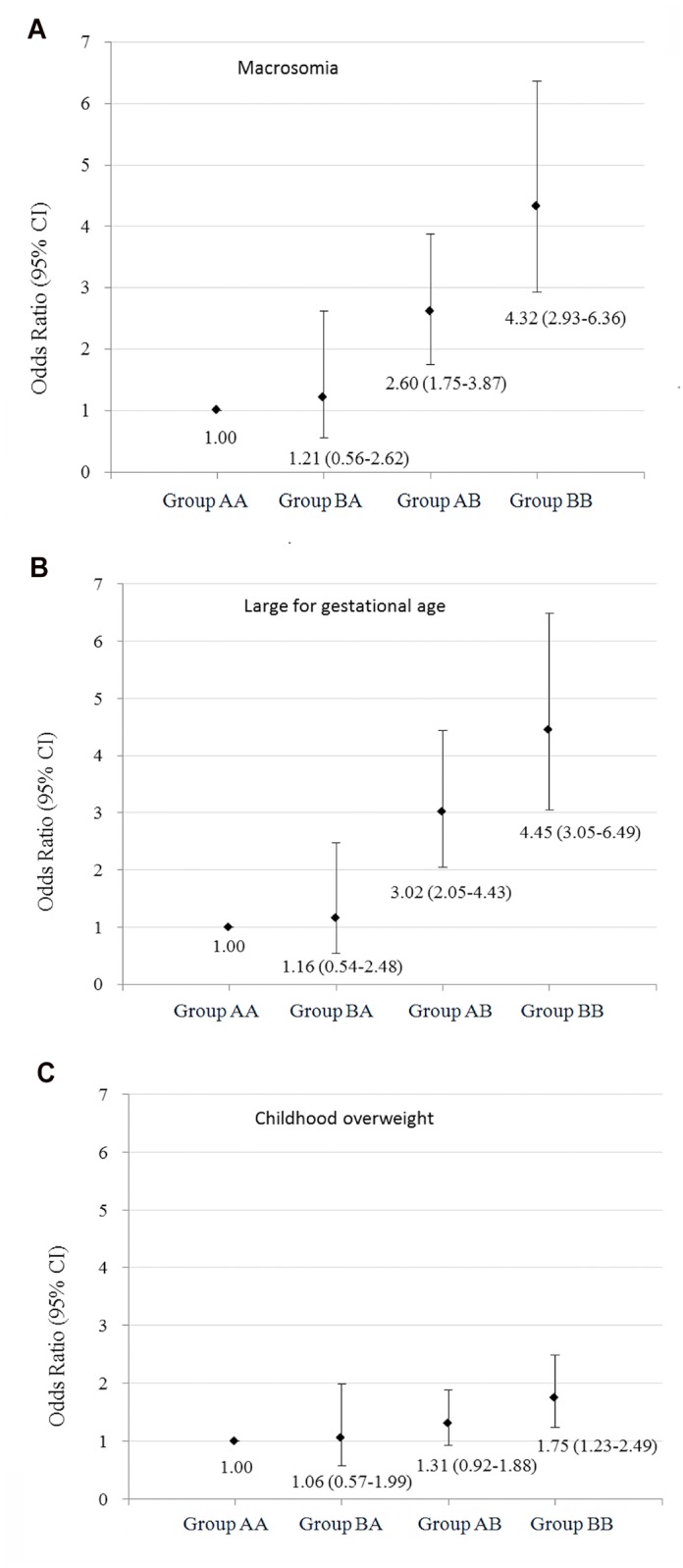
Odds ratios (95% CIs) of large for gestational age and macrosomia at birth and overweight at 1–5 years old according to joint status of maternal pre-pregnancy body mass index and gestational weight gain.

We additionally examined the joint association of maternal pre-pregnancy BMI and GWG with their offspring overweight status from birth to 1–5 years old by stratifying GDM mothers into four groups: pre-pregnancy normal weight (BMI <24 kg/m^2^) and non-excessive GWG, pre-pregnancy overweight (BMI ≥24 kg/m^2^) and non-excessive GWG, pre-pregnancy normal weight and excessive GWG, as well as pre-pregnancy overweight and excessive GWG (Table 4 and S3 Table). The positive association between pre-pregnancy BMI and the risks of large for gestational age and macrosomia at birth, and childhood overweight at baseline was found among GDM women with non-excessive GWG and excessive GWG. Similarly, the positive association between maternal GWG and the risks of large for gestational age and macrosomia at birth, and childhood overweight at baseline was found among GDM women with pre-pregnancy normal weight and overweight. Compared with offspring born to GDM mothers with pre-pregnancy normal weight and non-excessive GWG, offspring born to GDM mothers with pre-pregnancy overweight and excessive GWG had the highest risks of large for gestational age [ORs 95% CIs = 4.45 (3.05–6.49)] and macrosomia [ORs 95% CIs = 4.32 (2.93–6.36)] at birth and childhood overweight at 1–5 years old [ORs 95% CIs = 1.75 (1.23–2.49)], offspring born to GDM mothers with pre-pregnancy normal weight and excessive GWG had the higher risks of large for gestational age [ORs 95% CIs = 3.02 (2.05–4.46)] and macrosomia [ORs 95% CIs = 2.60 (1.75–3.87)] at birth and marginal higher risk of childhood overweight at 1–5 years old [ORs 95% CIs = 1.31 (0.92–1.88)] (Fig 2).

**Table 4:** Neonatal and childhood major outcomes at birth and 1–5 years old according to joint status of maternal pre-pregnancy body mass index and gestational weight gain.

	Maternal pre-pregnancy BMI (kg/m^2^) and gestational weight gain				P_overall_
	Pre-pregnancy BMI <24/non-excessive gestational weight gain (Group AA)	Pre-pregnancy BMI ≥24/non-excessive gestational weight gain (Group BA)	Pre-pregnancy BMI <24/excessive gestational weight gain (Group AB)	Pre-pregnancy BMI ≥24/excessive gestational weight gain (Group BB)	
No. of subjects	469	81	354	359	
**At birth** ^**a**^					
Birth weight for length for gestational age Z score	-0.09 (0.10)	-0.06 (0.15)	0.44 (0.11) ^#^ ^^^	0.62 (0.10) ^&^ ^†^ ^‡^	<0.001
**At 1–5 years old**					
Weight for length/height Z score					
Model 1 ^a^	0.30 (0.09)	0.48 (0.13)	0.53 (0.09) ^#^	0.72 (0.09) ^&^ ^†^ ^‡^	<0.001
Model 2 ^b^	0.38 (0.09)	0.54 (0.13)	0.52 (0.09)	0.68 (0.09) ^&^ ^‡^	<0.001
Body mass index for age Z score					
Model 1 ^a^	0.26 (0.09)	0.43 (0.14)	0.49 (0.10) ^#^	0.66 (0.09) ^&^ ^‡^	<0.001
Model 2 ^b^	0.33 (0.09)	0.49 (0.14)	0.48 (0.10) ^#^	0.62 (0.09) ^&^	0.001
Change in weight for height Z score from birth to 1–5 years old					
Model 1 ^a^	0.39 (0.12)	0.53 (0.18)	0.10 (0.13) ^#^ ^^^	0.11 (0.12) ^&^ ^†^	<0.001
Model 2 ^b^	0.03 (0.09)	0.19 (0.13)	0.17 (0.09)	0.32 (0.09) ^&^ ^‡^	<0.001

Data represent mean (SE), percentage or odd ratio (95% CI).

^a^, Adjusted for maternal age, family history of diabetes, education, family income, gestational diabetes treatment during pregnancy, gestational weeks of birth and infant feeding.

^b^, Adjusted for above variables and also birth weight for length for gestational age Z score.

* P <0.05 for Groups of AA and BA;

^#^ <0.05 for groups of AA and AB;

^&^ for groups of AA and BB;

^^^ P <0.05 for Groups of BA and AB;

^†^ <0.05 for groups of BA and BB;

^‡^ for groups of AB and BB.

## Discussion

The present study indicated that offspring born to GDM mothers with pre-pregnancy overweight/obesity or excessive GWG were associated with increased risks of large for gestational age and macrosomia at birth, which was consistent with previous studies [40–44]. Moreover, the present study found for the first time that pre-pregnancy overweight/obesity and excessive GWG of GDM mothers were positively associated with increased risks of childhood overweight of their offspring at 1–5 years old.

It has been previously shown that among the general population, maternal pre-pregnancy overweight/obesity and excessive GWG were associated with large birth weight and childhood overweight of their offspring [9–15]. However, at what age this association becomes apparent is unclear. Stunkard et al [45] suggested that the association of maternal pre-pregnancy overweight/obesity with childhood overweight did not emerge until at least 3–4 years of age. Li et al [15] showed that children born to obese mothers were twice as likely to be obese by 2 years of age. Another Chinese study from our team conducted in general population indicated that maternal pre-pregnancy overweight/obesity and excessive GWG was associated with increased risks of infancy (≤1 year of age) overweight and obesity [46]. To our knowledge, there were no corresponding studies targeting at the GDM women’s offspring aged younger than 5 years, and it was unclear how early in life children born to pre-pregnancy overweight/obesity and excessive GWG mothers with prior GDM began to express their risk of overweight. This study showed for the first time that maternal pre-pregnancy overweight/obesity and excessive GWG were positively associated with increased risks of childhood overweight at 1–5 years old.

We found that there were null or inverse associations of maternal pre-pregnancy overweight/obesity and excessive GWG with changes in weight for age Z score and weight for height Z score from birth to 1–5 years old. However, after additionally adjusted for birth weight for gestational age Z-score or birth weight for length for gestational age Z score, these associations became significant and positive. It was probably because birth size was another important risk factor for obesity in later life. Child born to mothers with pre-pregnancy overweight/obesity and excessive GWG seemed to grow faster in the first 1–5 years (change in z score for weight for height) compared with those born to mothers with pre-pregnancy normal weight and adequate GWG, after adjusted for birth size.

Our study also assessed the joint association of maternal pre-pregnancy BMI and GWG with the risks of offspring overweight at birth and 1–5 years old at baseline survey. We found that offspring born to GDM mothers with pre-pregnancy overweight/obesity and excessive GWG presented the highest risk of macrosomia and large for gestational age at birth and overweight at 1–5 years old compared with those born to GDM mothers with pre-pregnancy normal weight and adequate GWG. Another important observation was that the associations of maternal excessive GWG with macrosomia and large for gestational overweight of their offspring at birth were similar to that of maternal pre-pregnancy overweight/obesity, but the effects of maternal excessive GWG on their offspring’s overweight status at 1–5 years old were smaller than that of maternal pre-pregnancy overweight/obesity. This finding was confirmed in the subgroup analyses that the association of childhood overweight with maternal pre-pregnancy obesity tended to be larger among older children, while the association of childhood overweight with maternal excessive GWG tended to be smaller among older children. Previous studies suggested that among the general population, maternal pre-pregnancy overweight was a risk factor for both early onset overweight (persisted throughout childhood) and late onset overweight (after age 8) of their offspring, while maternal excessive GWG was only associated with the early onset overweight of their offspring [15]. Although offspring of mothers exposed to GDM are at increased risk of neonatal adiposity and childhood obesity after 5 years old, it is not clear whether the effect of GDM mothers with excessive GWG on offspring overweight will begin to attenuate with the growth of offspring. Thus, future studies are needed to answer this question.

In the present study, GDM women with pre-pregnancy obesity had a lower mean value of GWG. Since it has been suggested that pre-pregnancy obese women may benefit from low GWG [47], the IOM recommended lower optimal GWG for overweight/obese women. The 2009 IOM GWG recommendation has been widely used in Chinese pregnant women [37]. However, there were still a lot of women gaining more weight than the recommendation by the IOM guidelines (more than one third of pre-pregnancy normal weight GDM women and more than 50% of pre-pregnancy overweight/obese GDM women). Thus, a pregnancy lifestyle intervention is needed to control weight gain during pregnancy, especially for those who are pre-pregnancy overweight or obese.

There are several mechanisms that maternal pre-pregnancy overweight/obesity and excessive GWG might confer the risk of overweight/obesity of their offspring, including the child’s inheritance of genes susceptible to overweight/obesity [48], the intrauterine environment [49], and the maternal role in shaping the child’s postnatal eating and activity environment. Future studies are needed to distinguish among these possibilities.

Our study has several strengths, including use of GWG category instead of net weight gain, a large number of GDM mother-child pairs, and adjustment for multiple prenatal and perinatal factors in analyses. However, there were several limitations. First, the study was a retrospective cohort which might produce recall bias. Second, the participation rate was only 27%. Although there were no differences in age, 2-h glucose, fasting glucose, and the prevalence of IGT and diabetes at 26–30 weeks’ gestation (based on OGTT) between those returned and those not returned, whether there was a difference between the postpartum outcomes cannot be verified. Third, even though our analyses adjusted for an extensive set of confounding factors, residual confounding due to the measurement error in the assessment of confounding factors, unmeasured factors such as genetic and environmental factors of GDM mothers and their children, cannot be excluded.

## Conclusions

In summary, we found that offspring born to GDM mothers with pre-pregnancy overweight/obesity or excessive GWG had increased risks of large for gestational age and macrosomia at birth, and childhood overweight at 1–5 years old, compared with those born to GDM mothers with pre-pregnancy normal weight and adequate GWG.

## Supporting Information

S1 Table Recommendations for total weight gain during pregnancy by pre-pregnancy body mass index according to the Chinese maternal pre-pregnancy BMI classification standard and the 2009 IOM GWG recommendations. (DOCX)

S2 Table Mean values of Z scores at birth and 1–5 years old according to maternal pre-pregnancy body mass index and gestational weight gain categories. (DOCX)

S3 Table Mean values of Z scores at birth and 1–5 years old according to joint status of maternal pre-pregnancy body mass index and gestational weight gain categories. (DOCX)
