# Supplementary material for: GDM Women’s Pre-Pregnancy Overweight/Obesity and Gestational Weight Gain on Offspring Overweight Status
Source: PLoS One. 2015 Jun 22;10(6):e0129536. doi: 10.1371/journal.pone.0129536 (PMC4476720; doi:10.1371/journal.pone.0129536)
Supplement: S1 Table — (DOCX) [file pone.0129536.s001.docx]

**S1_Table.** Recommendations for total weight gain during pregnancy by pre-pregnancy body mass index according to the Chinese maternal pre-pregnancy BMI classification standard and the 2009 IOM GWG recommendations

| Pre-pregnancy BMI | Chinese pre-pregnancy BMI classification standard (kg/m^2^) | Total weight gain range (kg) |
| --- | --- | --- |
| Under weight | <18.5 | 12.5-18 |
| Normal weight | 18.5-23.9 kg/m^2^ | 11.5-16 |
| Overweight | 24.0-27.9 kg/m^2^ | 7-11.5 |
| Obese | ≥28.0 kg/m^2^ | 5-9 |

Adequacy of gestational weight gain (GWG) was classified according to the Chinese maternal pre-pregnancy BMI classification standard and the 2009 IOM GWG recommendations (above table); Inadequacy of GWG was defined as below adequacy of GWG and excessive of GWG was defined as above the adequacy of GWG.
