# Supplementary material for: GDM Women’s Pre-Pregnancy Overweight/Obesity and Gestational Weight Gain on Offspring Overweight Status
Source: PLoS One. 2015 Jun 22;10(6):e0129536. doi: 10.1371/journal.pone.0129536 (PMC4476720; doi:10.1371/journal.pone.0129536)
Supplement: S2 Table — (DOCX) [file pone.0129536.s002.docx]

**S2_Table.** Mean values of Z scores at birth and 1-5 years old according to maternal pre-pregnancy body mass index and gestational weight gain categories.

|  | **Pre-pregnancy BMI (kg/m^2^)** | | | **P_overall_** | **IOM categories** | | | **P_overall_** |
| --- | --- | --- | --- | --- | --- | --- | --- | --- |
|  | **<24**  **(Group A)** | **24-27.9**  **(Group B)** | **≥28**  **(Group C)** |  | **Inadequate**  **(Group D)** | **Adequate**  **(Group E)** | **Excessive**  **(Group F)** |  |
| No. of subjects | 823 | 335 | 105 |  | 156 | 394 | 713 |  |
| **At birth** ^a^ |  |  |  |  |  |  |  |  |
| Birth weight for gestational age Z score | 0.15 (0.09) | 0.48 (0.10) ^*^ | 0.76 (0.14) ^# ^^ | <0.001 | -0.25 (0.12) ^&^ | 0.09 (0.10) | 0.64 (0.09)^† ‡^ | <0.001 |
| Birth weight for length for gestational age Z score | 0.09 (0.09) | 0.38 (0.10) ^*^ | 0.69 (0.14) ^# ^^ | <0.001 | -0.32 (0.12) ^&^ | 0.04 (0.10) | 0.55 (0.09) ^† ‡^ | <0.001 |
| **At 1-5 years old** |  |  |  |  |  |  |  |  |
| Weight for age Z score |  |  |  |  |  |  |  |  |
| Model 1 ^a^ | 0.62 (0.08) | 0.85 (0.09) ^*^ | 1.07 (0.12) ^# ^^ | <0.001 | 0.56 (0.10) | 0.60 (0.09) | 0.89 (0.08) ^† ‡^ | <0.001 |
| Model 2 ^b^ | 0.69 (0.08) | 0.83 (0.08) ^*^ | 0.98 (0.11) ^#^ | 0.003 | 0.72 (0.10) | 0.68 (0.08) | 0.83 (0.08) ^†^ | 0.043 |
| Length/height for age Z score |  |  |  |  |  |  |  |  |
| Model 1 ^a^ | 0.64 (0.08) | 0.78 (0.09) ^*^ | 0.87 (0.12) ^#^ | 0.019 | 0.59 (0.10) | 0.64 (0.09) | 0.78 (0.08) ^† ‡^ | 0.017 |
| Model 2 ^b^ | 0.69 (0.08) | 0.76 (0.08) | 0.79 (0.11) | 0.43 | 0.73 (0.10) | 0.71 (0.08) | 0.73 (0.08) | 0.95 |
| Change in weight for age Z score from birth to 1-5 years old |  |  |  |  |  |  |  |  |
| Model 1 ^a^ | 0.47 (0.10) | 0.37 (0.11) | 0.31 (0.15) | 0.27 | 0.80 (0.13) ^&^ | 0.51 (0.11) | 0.24 (0.10) ^† ‡^ | <0.001 |
| Model 2 ^b^ | 0.28 (0.08) | 0.42 (0.08) ^*^ | 0.57 (0.11) ^#^ | 0.003 | 0.31 (0.10) | 0.27 (0.08) | 0.42 (0.08) ^†^ | 0.043 |

Data represent mean (SE), or percentage.

^a^ Adjusted for maternal age, family history of diabetes, education, family income, gestational diabetes treatment during pregnancy, gestational weeks of birth and infant feeding.

^b^ Adjusted for above variables and also birth weight for gestational age Z-score.

^*^ P <0.05 for Groups of A and B; ^#^ P<0.05 for groups of A and C; ^^^ P<0.05 for groups of B and C; ^&^ P for groups of D and E; ^†^ P <0.05 for Groups of E and F; ^‡^ P<0.05 for groups of D and F.
