# Supplementary material for: GDM Women’s Pre-Pregnancy Overweight/Obesity and Gestational Weight Gain on Offspring Overweight Status
Source: PLoS One. 2015 Jun 22;10(6):e0129536. doi: 10.1371/journal.pone.0129536 (PMC4476720; doi:10.1371/journal.pone.0129536)
Supplement: S3 Table — (DOCX) [file pone.0129536.s003.docx]

**S3_Table.** Mean values of Z scores at birth and 1-5 years old according to joint status of maternal pre-pregnancy body mass index and gestational weight gain categories.

|  | Maternal pre-pregnancy BMI (kg/m^2^) and gestational weight gain | | | | P**_overall_** |
| --- | --- | --- | --- | --- | --- |
|  | Pre-pregnancy BMI <24  /non-excessive gestational weight gain  (Group AA) | Pre-pregnancy BMI ≥24  /non-excessive gestational weight gain  (Group BA) | Pre-pregnancy BMI <24  /non-excessive gestational weight gain  (Group AB) | Pre-pregnancy BMI ≥24  /non-excessive gestational weight gain  (Group BB) |  |
| No. of subjects | 469 | 81 | 354 | 359 |  |
| **At birth ^a^** |  |  |  |  |  |
| Birth weight for gestational age Z score | -0.05 (0.10) | 0.05 (0.15) | 0.53 (0.11) ^# ^^ | 0.71 (0.10) ^&†‡^ | <0.001 |
| **At 1-5 years old** |  |  |  |  |  |
| Weight for age Z score |  |  |  |  |  |
| Model 1 ^a^ | 0.54 (0.08) | 0.74 (0.13) | 0.78 (0.09) ^#^ | 0.96 (0.09) ^&‡^ | <0.001 |
| Model 2 ^b^ | 0.65 (0.08) | 0.83 (0.12) | 0.75 (0.09) | 0.88 (0.08) ^&^ | 0.002 |
| Length/height for age Z score |  |  |  |  |  |
| Model 1 ^a^ | 0.59 (0.08) | 0.74 (0.13) | 0.72 (0.09) | 0.82 (0.09) ^&^ | 0.012 |
| Model 2 ^b^ | 0.69 (0.08) | 0.82 (0.13) | 0.70 (0.09) | 0.76 (0.08) | 0.57 |
| Change in weight for age Z score from birth to 1-5 years old |  |  |  |  |  |
| Model 1 ^a^ | 0.59 (0.11) | 0.69 (0.17) | 0.25 (0.12) ^# ^^ | 0.24 (0.11) ^&†^ | <0.001 |
| Model 2 ^b^ | 0.24 (0.08) | 0.42 (0.12) | 0.34 (0.09) | 0.48 (0.08) ^&^ | 0.007 |

Data represent mean (SE), or percentage.

^a^ Adjusted for maternal age, family history of diabetes, education, family income, gestational diabetes treatment during pregnancy, gestational weeks of birth and infant feeding.

^b^ Adjusted for above variables and also birth weight for gestational age Z-score.

^*^ P <0.05 for Groups of AA and BA; ^#^ <0.05 for groups of AA and AB; ^&^ for groups of AA and BB; ^^^ P <0.05 for Groups of BA and AB; ^†^ <0.05 for groups of BA and BB; ‡ for groups of AB and BB.
